# Cadge, of Norwich

**Published:** 1899-12-30

**Authors:** 


### "CADGE, OF NORWICH."

The death of Mr. William Cadge, F.R.C.S., so long and so honourably known throughout the Eastern Counties as a consulting surgeon, severs another link with the past. Mr. Cadge served his articles in the Norfolk and Norwich Hospital, after which he went up to University College Hospital, where he studied in the days of Liston, whose private assistant he became on passing the College of Surgeons, and so remained until Mr. Liston's death in 1847. He then became demonstrator of anatomy in University College, and in 1850 was appointed assistant surgeon to the hospital. This appointment, however, he did not hold long, for in a couple of years, a vacancy occurring on the staff of the Norfolk and Norwich Hospital, he returned to his old home and settled in Norwich, where for a long series of years he carried on a most successful practice, being called in consultation to all parts of the Eastern Counties, and earning high fame as an operating surgeon. "Cadge, of Norwich," has, indeed, been a household word in the counties of Norfolk and Suffolk for nearly half a century. It was, however, as an operator for stone in the bladder that he was best known in medical circles outside his own district. Perhaps no one man in England performed lithotomy so often or so successfully as did Mr. Cadge, whose record in this respect was unique. In 1874 he gave the address in Surgery to the British Medical Association, and in 1886 he was Hunterian Professor of Surgery, when he recounted before the Royal College of Surgeons his experiences in lithotomy and lithotrity. As the result of his operative work the museum of the Norfolk and Norwich Hospital has been enriched with a collection of specimens of calculus such as perhaps can never again be obtained by any museum, since the operation of lithotrity has so largely taken the place of the older operation of "cutting." Mr. Cadge never forgot the claims of the hospital in which he had been able by his genius and his industry to build up for himself the high position to which he attained. The Norfolk and Norwich Hospital and its requirements always occupied a central place in his thoughts, and twice he gave the munificent donation of £10,000 to its funds. He died at Lowestoft at the age of 75 years.

